# Using Digital Human Modelling to Evaluate the Risk of Musculoskeletal Injury for Workers in the Healthcare Industry

**DOI:** 10.3390/s23052781

**Published:** 2023-03-03

**Authors:** Xiaoxu Ji, Ranuki O. Hettiarachchige, Alexa L. E. Littman, Davide Piovesan

**Affiliations:** Biomedical Engineering, Gannon University, Erie, PA 16541, USA

**Keywords:** healthcare, injury, lower back pain, patient transfer, digital human modelling

## Abstract

Background: Hospital nurses and caregivers are reported to have the highest number of workplace injuries every year, which directly leads to missed days of work, a large amount of compensation costs, and staff shortage issues in the healthcare industry. Hence, this research study provides a new technique to evaluate the risk of injuries for healthcare workers using a combination of unobtrusive wearable devices and digital human technology. The seamless integration of JACK Siemens software and the Xsens motion tracking system was used to determine awkward postures adopted for patient transfer tasks. This technique allows for continuous monitoring of the healthcare worker’s movement which can be obtained in the field. Methods: Thirty-three participants underwent two common tasks: moving a patient manikin from a lying position to a sitting position in bed and transferring the manikin from a bed to a wheelchair. By identifying, in these daily repetitive patient-transfer tasks, potential inappropriate postures that can be conducive to excessive load on the lumbar spine, a real-time monitoring process can be devised to adjust them, accounting for the effect of fatigue. Experimental Result: From the results, we identified a significant difference in spinal forces exerted on the lower back between genders at different operational heights. Additionally, we revealed the main anthropometric variables (e.g., trunk and hip motions) that are having a large impact on potential lower back injury. Conclusions: These results will lead to implementation of training techniques and improvements in working environment design to effectively reduce the number of healthcare workers experiencing lower back pain, which can be conducive to fewer workers leaving the healthcare industry, better patient satisfaction and reduction of healthcare costs.

## 1. Introduction

Musculoskeletal disorders (MSDs) are one of the leading causes of disability in hospital nurses and caregivers as they have the highest number of reported occupational injury cases every year in the United States [1]. In 2021, overexertion as the major contributor caused 43.5% to 64.5% of back injuries in the healthcare industry [2]. In the same year, 300 nurses were involved in a self-administered survey in which 97.3% reported work-related pains, and 86.7% had the worst pain in their lower back [3]. In 2020, there were more than 175,000 cases of days away from work reported by nursing assistants and registered nurses [4]. In 2019, the Liberty Mutual Workplace Safety Index reported that the healthcare industry was one of the most severe workplaces with a high proportion of workplace injuries. An estimated cost of USD 1.77 billion was caused by overexertion and outside sources [5]. In the same year, out of 121 questionnaires distributed to nurses and caregivers, approximately half of the respondents reported upper and lower back problems [6]. Furthermore, the healthcare workers, including nursing assistants and registered nurses, reported that over 23,000 days were missed from work due to MSDs in 2018 [7].

Caregivers’ frequency and severity of injury are associated with certain types of patient-handling tasks. Among the riskiest tasks are moving patients from one bed to another, repositioning patients in bed, transferring patients from a wheelchair to a toilet/bathtub, or lifting patients from the floor [8]. Healthcare settings cause caregivers to adopt postures that are significantly different between individuals due to the lack of workstation customization that generally occurs in other industries. Patients’ weight is another factor affecting the forces on the caregivers’ lower back. The heavier the patients, the larger the load on the caregivers’ backs and shoulders, eventually leading to MSDs [9]. The lack of lifting equipment, shortages in staff [10], and prolonged exposure to a large trunk inclination angle [11] were other reasons leading to healthcare workers’ high risk of injury.

Numerous approaches have been implemented to reduce low back pain in healthcare workers. For example, manual handling training, stress management and stretching exercises were introduced as treatment options in [12]. Yet, there was no strong evidence for any intervention in preventing MSDs in nurses. According to a study questionnaire [10], training in the use of lifting aids has not been widely implemented. In addition, due to staff shortages, 72.6% of nurses still worked 12 h shifts. These can all be conducive to a high prevalence of MSDs in the lower back. Some materials also inform healthcare workers to avoid postures and movements that can cause injury [13,14]. However, these methods seem ineffective since the number of MSD cases has been rising [4]. Finally, since more nurses and nursing assistants are leaving the healthcare industry, employees have been unable to reduce their long shift hours.

Currently, digital human modelling (DHM) technology is prevalently implemented to avoid injuries in the workplace. For example, DHM can be used to enhance productivity by reducing the risk of incurring MSDs [15], simulate a variety of tasks at workplaces to improve organizational ergonomics by analyzing physical fatigue [16], and evaluate safety in public places to make it compatible with the elderly [17]. Yet, there is a lack of DHM posture study in the healthcare sector. The previous studies [18,19,20,21] were limited to single static pose analysis, which cannot imitate realistic human motion to fully estimate the potential risk occurring in workplaces. Moreover, the analysis of spinal load and the load capability of each anatomical joint is sensitive to the DHM-adopted postures [22]. DHM software does not usually implement any principle or algorithm for accurately predicting human movement [23]. Hence, creating each task by positioning individual DHM postures and later merging all postures to form one dynamic simulation remains the most common strategy [24].

DHM technology can be integrated with advanced motion-tracking systems to evaluate the risk of injury. The known limitations of magnetic-based and camera-based motion tracking technologies can be overcome by wearable inertial measurement units (IMU) [25]. The operational range of IMUs is quite extensive as it only depends on the range of wireless links [26]. IMUs do not require any fixed apparatus in the proximity of the working area, such as cameras or magnetic emitters. Hence, the application of IMUs within the workplace includes surgery [27], sports practice [28], and use on the factory floor [29]. Wearable IMUs are ideal for limited workspaces and unstructured environments (such as hospitals), as the body-worn tracking sensors are unobtrusive [26]. These systems do not limit body movement while performing tasks involving full body motion, presenting a clear advantage against infrared markers [30]. Yet, IMUs are known for their inherent drift that can be linear or quadratic depending on the order of the integration. Wearable tracking systems are usually a combination of accelerometers (second order integration to obtain position), gyroscope (first order of integration) and magnetometers [26]. Sensor-fusion techniques can be used to alleviate the drift problem. 

The Xsens system combines the signals from 3D gyroscopes, accelerometers and magnetometers to create accurate and drift-free orientation estimation for inertial sensors. Magnetic sensors are particularly important as they provide stability in the horizontal plane by sensing the Earth’s magnetic field’s direction like a compass. These complementary sensors combined with a Kalman filter reduce the drift by continuous correction of the orientation obtained by integrating sensor data.

This study combines the accuracy of wearable sensors in measuring full-body postures with the versatility of DHM to provide accurate ergonomics analysis. Although some common tasks in the healthcare sector have been assessed with this integrative approach in [8], more repetitive patient-transfer tasks need to be studied to help healthcare workers understand the awkward postures that must be avoided. This study focuses on lower back analysis and maximum hand force, which may lead to injuries when the lower back force reaches a safety threshold limit.

## 2. Methods

### 2.1. System Setup and Participants

This experimental study involved thirty-three participants in performing patient transfer tasks. The following data represent the average and standard deviation for each gender population (seventeen males: height 180.6 (10.6) cm, weight 83.4 (15.1) kg; sixteen females: height 165.7 (7.7) cm, weight 65.5 (10.8) kg). A total of seventeen Xsens MVN Awinda system wearable inertial sensors (Xsens 3D Motion Tracking Technology, Netherlands) were secured on each participant following the user manual. For each individual, we created a skeletal model (DHM_Xsens) in the Xsens software, which was used to record full-body movements. The sensors of the Xsens system were also used to establish the parameters for the digital model. The model accounts for (1) shoulder width, (2) hip widths, (3) arm span, (4) ankle height, and two values for the length of (5) upper arms, (6) lower arms, (7) hands, (8) thighs, (9) shanks and (10) feet.

JACK Siemens software integrates a wide range of population anthropometric data, human performance and motion prediction models [31]; it has been prevalently used in a variety of research areas [32,33,34] which extensively validated the force output. JACK also has the unique capability of integrating with the “Xsens MVN Analyze tool”; a piece of software used for ergonomic analysis. The skeletal roots providing the joint centroid positions in the cartesian space of the DHM_Xsens were imported into JACK software by setting up a unique port number in Network Streamer. By doing so, we created a second model for each participant in JACK (version C6.1), which we will indicate as DHM_JACK hereafter. The anatomical joint centers of DHM_JACK were properly aligned to the Xsens skeletal segments by using a scaling feature. This operation guarantees that the DHM_JACK will exactly copy any movement performed by the DHM_Xsens. This advanced integrative approach greatly eliminates the time required to create a full-body dynamic simulation by manually positioning each DHM anatomical joint.

### 2.2. Operational Tasks

Each participant performed two common patient transfer tasks that are repeated by healthcare workers daily. Participants followed the operational details determined by the UPMC Health System Nursing Assistant Orientation, to ensure basic consistency in task performance. 

**Figure 1:**
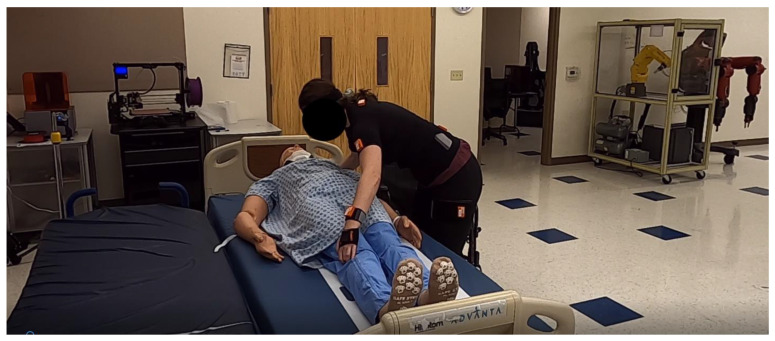
Task#1: Moving the patient manikin from a lying position to a sitting position in bed.

Task#1: A 25 kg patient manikin was located on the center of a hospital bed (Hill-Rom Advanta). Each participant was required to perform a lying-to-sitting task in bed, as shown in Figure 1. This task involved the following steps: (1) leaning the upper body forward, (2) extending both arms, (3) placing the right hand under the manikin’s head, (4) holding the manikin’s leg using the left hand, then applying force on both hands to move the manikin from a lying position to a sitting position on the edge of the bed. 

**Figure 2:**
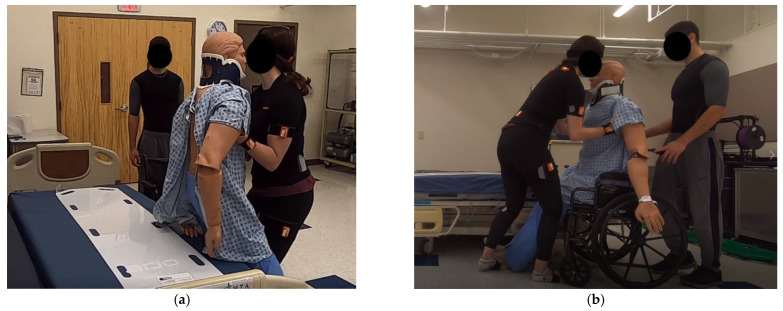
Task#2: Transferring the patient manikin from a bed to a wheelchair. (**a**) Moving the manikin from the hospital bed. (**b**) Placing the manikin into the wheelchair.

Task#2: Each participant was required to transfer the patient manikin from the edge of a hospital bed to a wheelchair (Everest & Jennings Product), as shown in Figure 2. This task involved the following steps: (1) leaning the body forward, (2) lifting the manikin up to a standing position by applying an upward force on the underarms of the manikin, (3) firmly holding the manikin straight, (4) moving the manikin in front of the wheelchair, and (5) gently placing the manikin onto the chair.

Two different bed operational heights (higher height: 32.5 in; lower height: 25.5 in) were designed. Each participant was required to perform four cycles executing Task#1 and Task#2 consecutively at each operational height, to ensure the reliability of results while minimizing fatigue.

### 2.3. Data Analysis

The forces generated on the 4th/5th lumbar spine (L4/L5) were analyzed using the Task Analysis Toolkit (TAT) in JACK. The application can estimate the compressive and anterior/posterior (A/P) shear forces based on the posture assumed by the model and the force generated at the hands. The magnitude, application point and direction of the force applied by each hand was added to the Human Control Panel. In this study, we focused on three specific postures which placed the healthcare workers at a higher risk of injury due to the excessive estimated forces (compressive and A/P) acting on the lower back. 

**Figure 3:**
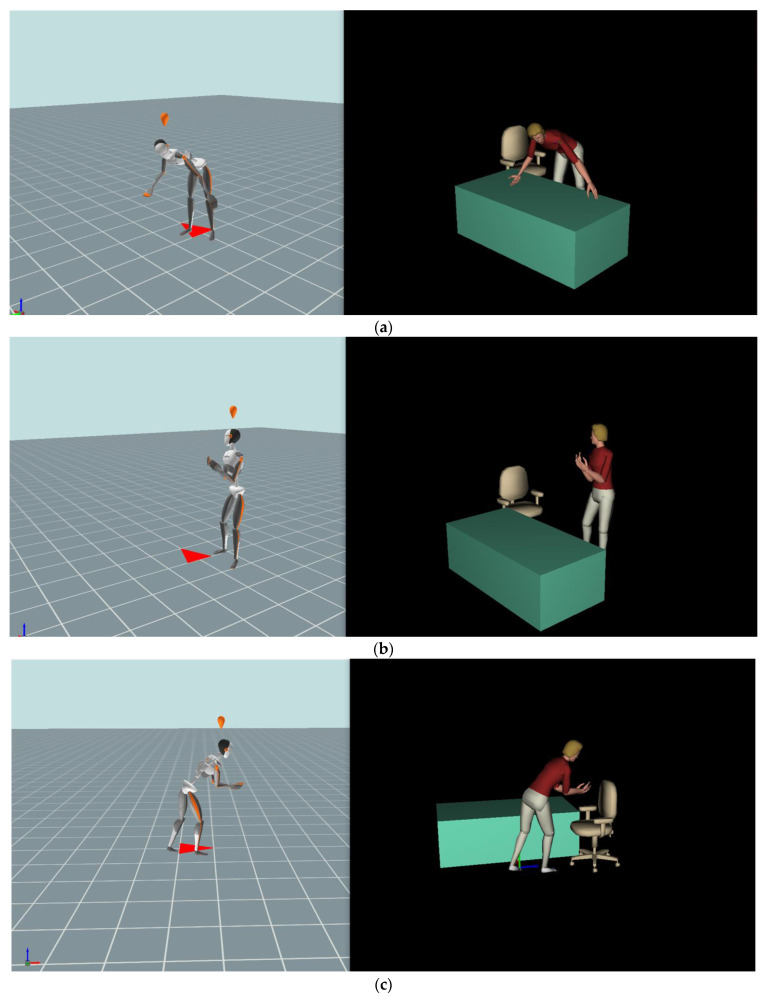
Three poses for the force analysis exposed on the lower back. The DHM_Xsens on the left represents the recorded actual human movement. The DHM_JACK on the right is driven by the DHM_Xsens. (**a**) Pose#1 in Task#1. (**b**) Pose#2 in Task#2. (**c**) Pose#3 in Task#2.

Pose#1: In the task of moving the patient manikin from a lying position to a sitting position (Task#1), the maximum spinal load occurred as the participants began to raise the head and upper trunk of the manikin in bed, as shown in Figure 3a. The force was measured by a digital force gauge (SF-500).

In the task of transferring the mock patient from the bed to the wheelchair (Task#2), excessive forces occurred at two poses. 

Pose#2: The participants stood toward the patient manikin with extended arms, flexed elbows, hands placed under the patient’s arms and bent upper body slightly backward to support the body weight of the manikin, as shown in Figure 3b.

Pose#3: In the same task of transferring the patient from the bed to the wheelchair (Task#2), the second at-risk pose was detected as the participants bent their upper body forward and extended both arms to place the patient manikin into the wheelchair, as shown in Figure 3c.

Finally, each hand’s maximum force exerted to reach a 3400 N compressive lower-back load was estimated at each of the three aforementioned poses. The National Institute for Occupational Safety and Health (NIOSH) [35] suggested the aforementioned spinal compressive load as the at risk value for developing MSDs. Once the estimated hand force is determined using the biomechanical module in JACK, healthcare workers will understand the maximum force they can exert during their daily patient transfer tasks to avoid the risk of injury.

### 2.4. Statistical Analysis

The data distribution for each of the variables (compressive force, A/P shear force, trunk and hips) will be tested by using MATLB (MathWorks Inc., Monterey, CA, USA). At each pose, a two-way analysis of variance (ANOVA) was performed to determine the significant difference between genders and two operational heights for each of the aforementioned variables. To reduce the chance of failure, the Bonferroni correction was involved to adjust the *p* values when several independent or dependent variables are tested simultaneously on a single data set. Considering that the calculated *p* values for some significantly different variables were too small, even after the Bonferroni correction post hoc analysis, the conclusion to describe the significance was not changed. Additionally, to fully understand the impact of each variable, a *t*-test was performed to analyze the compressive force, A/P shear force and the joint angular displacement between genders at each of the operational heights. Hence, the *p* values for each independent *t*-test are presented in this study. The statistically significant level was set at 0.05. Moreover, we analyzed the cross-correlation (R) between the spinal forces and either body height, weight or joint angles. Considering the significance of the correlation coefficient is highly related to the sample size (thirty-three participants in this study) [36], the critical values are ±0.35, indicating that if the calculated R values are greater than +0.35 or less than −0.35, they are significant. 

## 3. Results

The normality of the distribution for each of the variables (compressive force, A/P shear force, trunk and hips) was confirmed using a Kolmogorov–Smirnov test. All the *p* values were found to be less than 3.2 × 10^−7^, which rejected the null hypothesis at the 5% significance level. 

The settings at each of the specific postures to estimate the ergonomics results are listed in Table 1. The spinal forces acting on the lower back and the estimated anatomical joint angles are listed in Table 2 and Table 3. The correlation coefficients for both compressive and A/P loads are listed in Table 4. The explanation for the calculated results at each pose is described in the following Section 3.1, Section 3.2 and Section 3.3, respectively.

### 3.1. Pose#1 in Task#1: Assisting Patient Manikin from Lying to Sitting in the Hospital Bed

#### 3.1.1. Force Analysis

In Task#1, the measured force magnitude applied on the right hand was approximately 70 N. The direction was vertically upward. At this time frame, the force applied by the left hand could be consider negligible.

**Figure 4:**
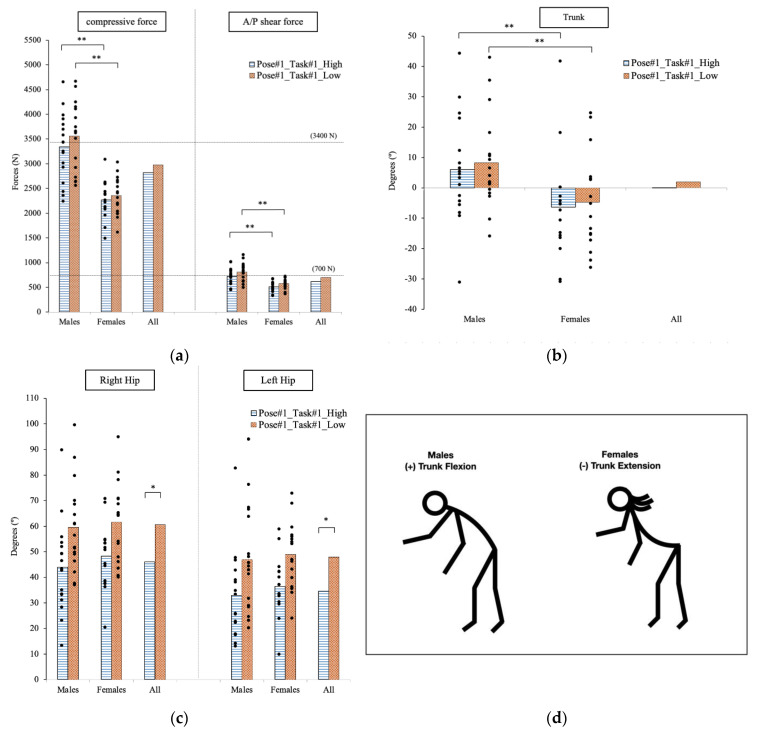
At Pose#1 in Task#1, the forces exposed on the lower back at two operational heights are plotted in (**a**). The trunk joint is plotted in (**b**). The hip joints are plotted in (**c**). Stick figures illustrating the significant difference in postures for males and females are shown in (**d**). * indicates a significant difference between two operational heights. ** indicates a significant difference between genders.

The maximum forces exerted on the lower back occurred at Pose#1 when the participant bent over at the waist, the right hand supported the manikin’s head, the left hand held the legs, and the participant started raising the manikin’s head and trunk from the bed. There was no significant difference in the spinal load of the participants as they exerted the lifting effort at two different operational heights (*p* > 0.05, see Figure 4a). However, the spinal forces estimated on the male participants were statistically larger than the female participants for the compressive force analysis with *p* < 0.0001 (males: 3342.6 N; females: 2266.7 N), and for the A/P shear force with *p* = 0.0002 (males: 720.4 N; females: 510.9 N) at the higher operational height. Furthermore, the *p* values were less than 0.0001 for the analysis of compressive force (males: 3559.8 N; females: 2360.3 N) and A/P shear force (males: 812.2 N; females: 574.1 N) at the lower operational height.

The maximum applied hand force was estimated until the compressive force’s safety threshold limit reached 3400 N at each specific pose. The estimated hand force was not significantly different at both operational heights, but it was statistically different between genders (*p* < 0.0001). For the female population, the predicted average forces exerted by each hand were 168.2 N when the bed was at 32.5 in, and 159.9 N when the bed was at 25.5 in. The maximum forces in the female population were more than two times greater than for the male population which reached only 82.4 N at the higher height and 69.8 N at the lower height.

#### 3.1.2. Joint Angle

There are two important joint centers defined in DHM_JACK. The Root joint is defined as the center of two greater trochanters. Spine#1 joint is defined as the center of two posterior superior iliac spine (PSIS). Hence, the trunk movement is based on the relatively angular displacement between Vector#1 (from the Acromion to Spine#1) and Vector#2 (from Spine#1 to the Root). The hip movement is based on the relatively angular displacement between Vector#1 (from Spine#1 to the Root) and Vector#2 (from the Root to the Knee).

The comparison of joint angular displacements is shown in Figure 4b,c. Assuming that the trunk angle and the hip angle are 0° at a neural erected pose, the positive and negative values for the trunk and hips indicate flexion and extension, respectively. The hip movement was significantly different at both operational heights (right hip: *p* = 0.0006; left hip: *p* = 0.0012). The average angular displacements of the right hip for the whole population were 44.8° and 60.6° at the higher and lower operational heights, respectively. For the left hip, the angular displacements were 34.1° and 47.9° for the higher and lower heights, respectively. Interestingly, despite the different size of the subjects, there was no significant difference between genders.

On the other hand, there was no significant difference between trunk angular displacement at both operational heights for all participants. Yet, the difference was statistically different between genders (higher height: *p* = 0.049; lower height: *p* = 0.024). At the higher operational height, the average trunk angles were 6.1° (flexion) for males, and −6.4° (extension) for females. At the lower operational height, the average trunk angles were 8.3° (flexion) for males, and −4.8° (extension) for females. 

#### 3.1.3. Cross-Correlation

There was a high correlation between body height and spinal forces. The R values were 0.93 and 0.86 for the compressive and A/P shear forces, respectively, at the higher height, and 0.94 and 0.91, respectively, at the lower height. There was also a high correlation between body weight and spinal forces. The R values were 0.78 and 0.72 for the compressive and A/P shear forces, respectively, at the higher height, and 0.79 and 0.80, respectively, at the lower height. The correlation between the trunk angular displacement and compressive force was moderate with the R values of 0.51 and 0.43 at the higher and lower heights, respectively. It was interesting to notice that there was a relatively high negative correlation between the trunk and hip movement with the average R values of −0.62 and −0.58 at the higher and lower operational heights, respectively.

### 3.2. Pose#2 in Task#2: Moving the Manikin from the Bed to the Wheelchair

#### 3.2.1. Force Analysis

The direction of the force applied by the participant to the patient was assumed vertically upward. Considering that both feet of the patient barely contacted the floor, the force distributed to each hand was approximately 125 N (25 kg × 9.8/h). When assuming h = 2 the force was equally distributed between the two hands. Although there was slight contact between the patient and the edge of the bed, the 125 N estimated force exerted by each hand is a good approximation to reveal the effect of key anthropometric variables affecting the force generated at the lower back of each participant.

**Figure 5:**
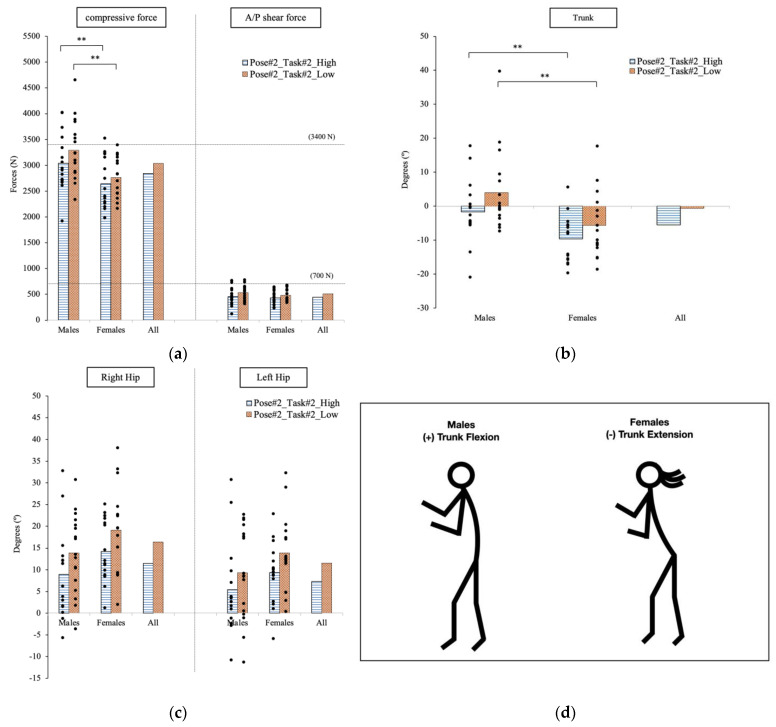
At Pose#2 in Task#2, the forces exposed on lower back at two operational heights are plotted in (**a**). The trunk joint is plotted in (**b**). The hip joints are plotted in (**c**). Stick figures illustrating the significant difference in postures for males and females are shown in (**d**). ** indicates a significant difference between genders.

The maximum spinal forces occurred at the pose when the participants put their hands under the arms of the manikin to start lifting from the bed (Figure 5a). The estimated compressive forces exerted on the lower back of males (higher height: 3038.1 N; lower height: 3293.2 N) were significantly different than the forces on the females’ back (higher height: 2644.1 N; lower height: 2766.2 N) at both operational heights (higher height: *p* = 0.041; lower height: *p* = 0.005).

To reach the safety threshold limit of 3400 N when raising the patient manikin from the bed at two different heights, the females can exert approximately more than 30 N force per hand than the males. At the higher operational height, the estimated force for females was 176.2 N per hand, as compared to 150.8 N for males. At the lower height, the average force for females was 166.0 N, and the force for males was 136.6 N.

#### 3.2.2. Joint Angle

When comparing the hip angular displacements, the right hip has 5° difference at the two operational heights (higher height: 11.4°; lower height: 16.4°). For the comparison between males and females, the trunk angular displacement was significantly different between the two groups with *p* = 0.008 at the higher height, and *p* = 0.015 at the lower height. In Figure 5b,c, the female population shows a greater trunk extension than the males in the patient transfer activity. At the higher operational height, the average trunk extension was −9.6° for the female group, and −1.7° for the male group. At the lower height, we find −5.6° (extension) for the females and 4.0° (flexion) for the males. 

#### 3.2.3. Cross-Correlation

The trunk and hip movements were still negatively correlated with the lumbar compression force at Pose#2 in Task#2. The estimated A/P shear force and the hip flexion were highly correlated at both heights (higher height: R = 0.68; lower height: R = 0.60). Additionally, the body weight was moderately correlated with the lumbar compressive force with R = 0.41 and R = 0.48 at the high and low operational height, respectively. The correlation between the compressive force with the body height were R = 0.49 at the higher height, and R = 0.68 at the lower height.

### 3.3. Pose#3 in Task#2: Positioning the Patient Manikin in Wheelchair

#### 3.3.1. Force Analysis

Considering that the excessive spinal force was estimated when the patient manikin just barely touched the wheelchair, each participant still held most of the manikin’s body weight. While the support from the manikin’s feet could reduce the loads applied by both hands, the 125 N estimated force to each palm was an approximated value which represents a figure of merit for the load. Given that end-users can easily modify the force values to represent the lifting efforts, having an approximated magnitude of the force would still provide useful information for a sensitivity analysis.

**Figure 6:**
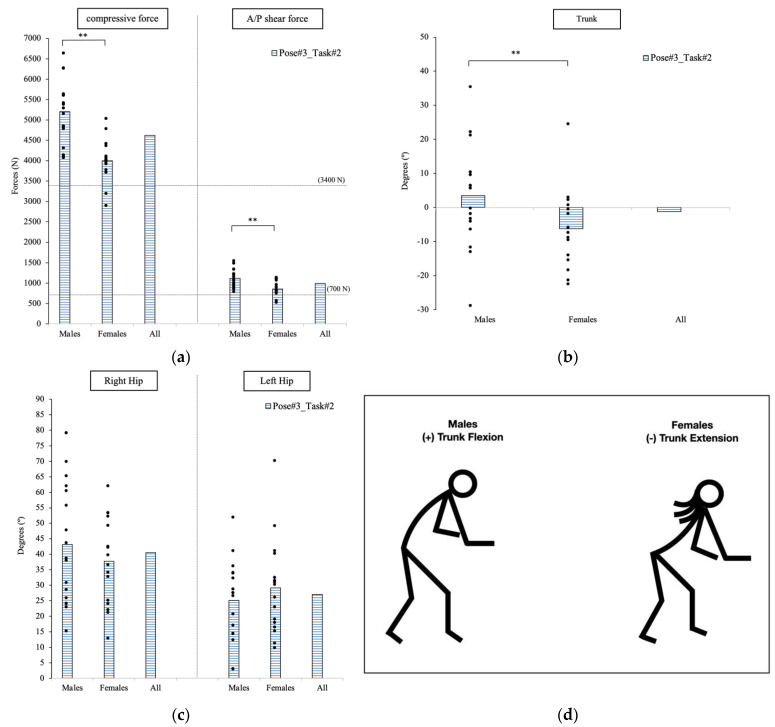
At Pose#3 in Task#2, the forces exposed on the lower back are plotted in (**a**). The trunk joint is plotted in (**b**). The hip joints are plotted in (**c**). Stick figures illustrating the significant difference in postures for males and females are shown in (**d**). ** indicates a significant difference between genders.

The second pose to have an excessive spinal force in Task#2 occurred as the participants moved the patient manikin into the wheelchair with a large hip flexion, as well as arm extension and knee flexion. The estimated spinal loads on the males were significantly greater than the forces on the females (*p* < 0.001; Figure 6a). The average compressive forces in males and females were 5201.0 N and 4003.3 N, respectively. The A/P shear force was also dangerously higher, averaging 1116.6 N in the male population and 854.7 N in the female population. Nearly all participants (31 out of 33) had a spinal load larger than the safety threshold limit of 3400 N for the compressive force [35], and 700 N for the shear force [37].

The hand force exerted by females to reach the compression safety threshold of 3400 N was nearly twice the males at this specific pose (*p* < 0.0001). The average safe applicable hand force was approximately 100 N per hand for females, and 50 N for males.

#### 3.3.2. Joint Angle

The trunk angular displacement was significantly different between genders with *p* = 0.047. Females extended their trunk (−6.2°) while male flexed it (3.5°) at this specific pose. There was no significant difference between hip angles between genders at this specific pose, as shown in Figure 6b,c.

#### 3.3.3. Cross-Correlation

Both body weight and height are highly correlated with the lower back compressive force with R = 0.65 and R = 0.81, respectively. Both anthropometric variables were also highly correlated with the A/P shear force with R = 0.59 and R = 0.71, respectively. Additionally, the A/P shear was highly correlated with the right hip flexion (R = 0.66). A high negative correlation between the movement of the trunk and hips was found with an average R = −0.58.

## 4. Discussion

In this study, the participants were required to perform two common patient transferring tasks. The spinal forces exerted on the lower back were assessed. During the entire task performance, three specific postures are assumed to provoke excessive force on the spinal column, which could put healthcare workers at risk of injury. At each of the aforementioned poses, we analyzed the effect of the bed operational height on the posture adoption for all participants and the effect of key anthropometric and biomechanical factors affecting the force on the lower back between genders.

As the operational bed height was changed from 32.5 in to 25.5 in at Pose#1, only the hip angular displacement was significantly different for all participants, which was consistent with the result in [8]. The spinal forces and the trunk movement were not noticeably different among participants between the two heights. The physical constraints of the adopted posture could be the cause. Participants needed to extend their arms to support the manikin’s head and leg to facilitate the transition of the patient from lying to sitting in bed. Meanwhile, the individual’s eyes concentrated on the manikin’s face. This action may involve the extension of the participant’s head, which may somewhat constrain their trunk flexion. This phenomenon is consistent with the result in [38].

When comparing the spinal forces between genders, the forces on the spinal column of males were significantly larger than their female counterparts. The difference in compressive force amounted to approximately 1000 N. Given the lifting force, the trunk and hip movement determined the load amplitude affecting the participants’ lower back [22,39]. Our statistical analysis revealed a moderate correlation between the trunk angular displacement and the compressive spinal force at two different operational heights. Moreover, a high negative correlation between the movement of hips and trunk existed at Pose#1, particularly among females. Due to their relatively short body height, females have to flex their hips, and extend their arms and trunk to reach the manikin. The difference in average body height (15 cm) allowed the males to simply flex their trunk instead of adequately extending their spine into the lifting task. Given this significant difference in posture adoption between genders, the force affecting the lower back of males quickly reached the safety threshold limit of 3400 N even if the force exerted by each hand is merely 70 N. We can foresee that healthcare workers lift larger loads daily, putting them at a high risk of injury during their daily activities. Accordingly, providing the proper training and ergonomic adjustability of the beds to avoid awkward postures (trunk flexion) are keys to preventing workers from incurring injury when transferring a patient from lying to sitting in bed.

At Pose#2, the movement of the right hip has 5° difference among the participants, as they started lifting the manikin from the bed at two operational heights. Considering the proper lifting techniques suggested in the patient transfer task [13,14], healthcare workers should stand closer to the patient to avoid large trunk flexion, as shown in Figure 2. In this case, the limited space between the participants and the manikin constrained the participants’ trunk movement, forcing them to flex their hips to start the transferring task. Additionally, due to the position of the wheelchair on the right side of the bed, each individual preferred to tilt their body to the right side while simultaneously extending the left lower limb. The aforementioned posture limited any significant difference of the left hip movement when the operational height was changed at Pose#2.

At Pose#2, the trunk angular displacement and the compressive spinal force were significantly different between genders, consistent with the results at Pose#1. Again, high correlation between the A/P shear force and the hip flexion was revealed. Although males and females have some difference in the anthropometric and biomechanics aspects (e.g., the width of hip span and the muscle strength), both genders have a similar range of motion of trunks [40]. Hence, to prevent back injuries, both females and males should keep the upper body straight, bend the knees, and engage lower body muscle groups to lift an object safely, as mentioned by the Mayo Clinic [41]. The estimated force exerted by each hand to reach the spinal force safety threshold was also quite different between genders. At the higher operational height, the average estimated force was 175 N per hand for females as compared to 150 N per hand for males. Hence, females would be exposed to the risk of injury when lifting a 35 kg load. On the other hand, for the male population, the risk threshold was reached by lifting a 30 kg load. As the operational height was changed to 25.5 in, the average hand force was reduced by 10 N for both genders. Accordingly, the lifting load should be commensurate to the operational height, and it should either be decreased at lower heights, or redistributed by recruiting more workers to help completing the task. These results could help healthcare workers avoid injury when the lifting load is approximately or greater than the estimated 30 kg load at this specific pose. 

At Pose#3, the spinal forces exerted by the participants (31 out of 33) were greater than the safety threshold limits for both the compressive force (3400 N) and shear force (700 N). Moreover, the forces exerted by the male population were significantly greater than the females. One major reason was the notable discrepancy of trunk movement. While the height of the person plays a big role, the perceived muscular strength is another important factor influencing individual postures [42]. Male participants preferred to position the patient manikin in the wheelchair by flexing their trunk, while females preferred to extend trunk and flex their hips. Indeed, the A/P shear force was highly correlated with the right hip flexion at this specific pose, because participants needed to step forward with their right foot to place the patient manikin in the wheelchair. Based on the maximum hand force analysis, males would reach a safety threshold when only 50 N was exerted by each hand while females could exert up to 100 N to put the healthcare worker at risk of injury. Accordingly, an appropriate assistive device or more workers should be involved to reduce the lifting load for healthcare workers.

At all three poses, both anthropometric variables, body height and weight, indicated a high correlation with the spinal forces, which was consistent with the results in previous studies [43,44]. More body weight supported by the lower trunk may lead to an increased load on the lower back. Considering that the carried load and the trunk flexion are key factors in the risk of lower back injury, the heavier and taller the workers are, the higher the risk is for them to incur MSDs during patient transfer tasks.

## 5. Conclusions

In this work we highlighted two distinct patient transfer tasks. We found that even though trained in the proper lifting techniques, a large force can be exerted on the lower back of healthcare workers as they assist a patient from lying to sitting in bed and moving them from the bed to a wheelchair. When the operational height of the bed is changed, the hip movement has a significant influence on posture adoption. Additionally, given the difference of anthropometric and biomechanics variables between genders, the male population is more easily exposed to a high risk of injury than females. Hence, for safety considerations, the lifting load and the adopted posture must be extremely controlled.

## Figures and Tables

**Table 1:** The settings at each of the specific postures to estimate the ergonomic results. “P” represents poses. “T” represents tasks.

	Operational Height	Force Magnitude	Application Point	Force Direction
P#1_T#1	high	70 N	right hand	vertical
	low	70 N	right hand	vertical
P#2_T#2	high	125 N	both hands	vertical
	low	125 N	both hands	vertical
P#3_T#2	--	125 N	both hands	vertical

**Table 2:** The estimated spinal forces at each of the specific postures. The results are presented as the mean value (standard deviation).

	Operational Height	Average Comp Force_Male	Average Comp Force_Female	Average A/P Force_Male	Average A/P Force_Female
P#1_T#1	high	3342.6 N (682.4 N)	2266.7 N (382.2)	720.4 N (173.8 N)	510.9 N (100.2 N)
	low	3559.8 N (686.7 N)	2360.3 N (375.5 N)	812.2 N (185.3 N)	574.1 N (103.0 N)
P#2_T#2	high	3038.1 N (549.0 N)	2644.1 N (511.4 N)	455.6 N (161.7 N)	432.5 N (133.0 N)
	low	3293.2 N (584.4 N)	2766.2 N (388.1 N)	534.2 N (140.1 N)	483.9 N (118.0 N)
P#3_T#2	--	5201.0 N (781.0 N)	4003.3 N (521.5 N)	1116.6 N (221.0 N)	854.7 N (168.9 N)

**Table 3:** The estimated joint angles at each of the specific postures. The results are presented as the mean value (standard deviation).

	Operational Height	Average Trunk Angle		Average Right Hip		Average Left Hip	
		Male	Female	Male	Female	Male	Female
P#1_T#1	high	6.1° (17.5°)	−6.4° (17.6°)	44.0° (17.8°)	48.3° (12.6°)	32.9° (16.9°)	36.5° (11.5°)
	low	8.3° (15.6°)	−4.8° (16.0°)	59.7° (17.3°)	61.6° (15.9°)	47.0° (20.8°)	49.0° (13.1°)
P#2_T#2	high	−1.7° (9.0°)	−9.6° (6.9°)	8.9° (9.9°)	14.2° (7.2°)	5.4° (10.1°)	9.3° (7.0°)
	low	4.0° (11.8°)	−5.6° (9.7°)	13.9° (9.1°)	19.2° (10.0°)	9.3° (10.6°)	13.9° (8.7°)
P#3_T#2	--	3.5° (15.0°)	−6.2° (11.6°)	43.2° (19.3°)	37.8° (14.1°)	25.1° (13.2°)	29.1° (15.7°)

**Table 4:** The correlation coefficients for both compressive and A/P loads. Values greater than 0.35 are bold.

Variable vs. Force		Body Height	Body Weight	Hip	Trunk
P#1_T#1 high	Comp	**0.93**	**0.78**	0.01	**0.51**
	A/P	**0.86**	**0.72**	0.27	0.34
P#1_T#1 low	Comp	**0.94**	**0.79**	0.09	**0.43**
	A/P	**0.91**	**0.80**	0.22	0.30
P#2_T#2 high	Comp	**0.49**	**0.41**	**0.47**	0.32
	A/P	0.26	0.22	**0.68**	0.18
P#2_T#2 low	Comp	**0.68**	**0.48**	0.21	0.34
	A/P	**0.43**	0.29	**0.60**	0.04
P#3_T#2	Comp	**0.81**	**0.65**	**0.43**	**0.37**
	A/P	**0.71**	**0.59**	**0.66**	0.15

## Data Availability

Not applicable.
